# Hemostasis system condition in progress of sepsis in severe burns

**DOI:** 10.1186/cc9867

**Published:** 2011-03-11

**Authors:** M Presnyakova, S Evstigneev

**Affiliations:** 1 Nizhny Novgorod Research Institute of Traumatology and Orthopedics, Nizhny Novgorod, Russia

## Introduction

Among infectious complications in patients with serious heat injury, the most dangerous is sepsis developing in the early stages of burn disease. Sepsis is characterized by a fulminant, severe course, complex diagnostics, and a high case fatality rate. Hemostasis system disorders are one of the key pathogenic links of organ failure development in sepsis.

## Methods

The hemostasis system condition was studied in 100 patients with over 20% of the body burned, from the first to 12th day after burn. Examined patients were divided into two groups: in the first group an acute period of burn disease was complicated by progress of sepsis (33 patients), and in the second group complications in the form of sepsis were not observed. The groups of patients studied were balanced in age and severity of the disease. Sepsis was diagnosed on the basis of clinical, laboratory and bacteriological findings, as well as confirmed by morphological studies in casualties. The control group consisted of 130 apparently healthy people.

## Results

Comparative analysis of hemostasis system disorders in severe burn patients with early sepsis and those without similar complication showed that the progress of generalized infection in the acute period of burn disease is accompanied by reliable decreased activity of antithrombin III, XIIa-dependent fibrinolysis, blood plate count, and prothrombin time prolongation. There were no differences revealed between the studied groups of severe burn when determining fibrinogen content, soluble fibrin monomeric complexes, activated partial thromboplastin time, thrombin clotting time and echitox time, and the test revealing fragmented erythrocytes. Correlation analysis showed that the most contingency between progress of sepsis and hemostasis system data was noted on the third to fourth days after burn (with decreased activity of XIIa-dependent fibrinolysis (*r *= 0.58, *P *< 0.0001), antithrombin III (*r *= -0.57, *P *< 0.0001), prothrombin time (*r *= 0.49, *P *< 0.0001) and thrombocytopenia (*r *= -0.48, *P *< 0.0001)). On the basis of a retrospective analysis of case histories of severe burns with verified generalized infection, it was determined that the development of an acute form of DIC syndrome manifesting in a marked imbalance of coagulation and anticoagulative blood mechanisms as well as severe hepatorenal failure has a lead time of 1 to 8 days in revealing sepsis in the clinic.

## Conclusions

Hemostasis system disorders corresponding to an acute form of DIC syndrome not only accompany the progress of sepsis in severe burn but can be an indirect predictor of its development.

